# Insulin Resistance Associated Disorders Pivoting Long-Term Hepatitis B Surface Antigen Decline During Entecavir Therapy

**DOI:** 10.3390/jcm8111892

**Published:** 2019-11-06

**Authors:** Tien-Ching Lin, Wen-Chun Liu, Yu-Hsiang Hsu, Jia-Jhen Lin, Yen-Cheng Chiu, Hung-Chih Chiu, Pin-Nan Cheng, Chiung-Yu Chen, Ting-Tsung Chang, I-Chin Wu

**Affiliations:** 1 Department of Internal Medicine, National Cheng Kung University Hospital, College of Medicine, National Cheng Kung University, Tainan 704, Taiwan; campbell6th@hotmail.com (T.-C.L.); ttc10008031@gmail.com (J.-J.L.); tannoy63352@gmail.com (Y.-C.C.); toad.chiu@gmail.com (H.-C.C.); pncheng@mail.ncku.edu.tw (P.-N.C.); chiungyu@mail.ncku.edu.tw (C.-Y.C.); ttchang@mail.ncku.edu.tw (T.-T.C.); 2 Institute of Clinical Medicine, College of Medicine, National Cheng Kung University, Tainan 704, Taiwan; brianhsu@mail.ncku.edu.tw; 3 Infectious Disease and Signaling Research Center, National Cheng Kung University, Tainan 701, Taiwan; graceliu8911@gmail.com; 4 Clinical Medicine Research Center, National Cheng Kung University Hospital, College of Medicine, National Cheng Kung University, Tainan 701, Taiwan

**Keywords:** chronic hepatitis B, nucleos(t)ide analogs, diabetes mellitus, fatty liver disease

## Abstract

Insulin resistance associated disorders (IRAD), including prediabetes, type 2 diabetes mellitus (T2DM), and fatty liver are significant risk factors of liver-related death in chronic hepatitis B (CHB). However, their relationship remains unclear. We aimed to evaluate how IRAD influence the kinetics of serum hepatitis B surface antigen (HBsAg) in patients with CHB during long-term entecavir treatment. We enrolled 140 patients with CHB receiving at least 3 years of consecutive entecavir treatment in this retrospective study. A linear mixed effects model was adopted to examine the effects of variables and their interaction over time on the HBsAg trajectory. Furthermore, we acquired cytokine profiles and baseline fibrosis-4 index (FIB-4) scores for in-depth analysis. The median treatment time was 6.90 (4.47–9.01) years. Multivariate analysis revealed that older patients or those with prediabetes or T2DM had a significantly slower HBsAg decline over time (*p* = 0.0001 and *p* < 0.0001, respectively). Conversely, advanced fatty liver engendered a more rapid HBsAg decrease (*p* = 0.001). Patients with prediabetes or T2DM possessed higher IP-10 levels six years after entecavir therapy (*p* = 0.013). Compared to patients without prediabetes or T2DM, diabetic patients had more unfavorable features at the baseline, especially higher FIB-4 scores. Prediabetes or T2DM delays the clearance of HBsAg, but advanced hepatic fatty change counterbalances the effect. Additionally, IRAD could cause hepatic sequelae in CHB through immune-metabolic pathways.

## 1. Introduction

Chronic hepatitis B virus (HBV) infection remains a leading cause of liver cirrhosis (LC) and hepatocellular carcinoma (HCC) globally [1]. Due to HBV’s covalently closed circular DNA and integrated HBV DNA sequences in infected hepatocytes, there is still a long way to go to cure chronic hepatitis B (CHB) [2]. Nevertheless, an approach to reduce HBV-related morbidity and mortality is available in the era of pegylated interferon-alfa (PegIFNa) and nucleos(t)ide analogs (NA) [1]. In addition to medical therapy, the interplay between host immunity and HBV plays a critical role in HBV control and related pathogenesis [3]. Host immunity greatly determines the chronicity, different phases, and treatment response of CHB [1,3]. The whole picture of how our immunity works is only partially understood, but many significant factors that affect the immune system have been reported, especially immune-metabolic interactions [4]. Therefore, regarding the overall long-standing consequences of CHB, three distinct, but mutually interacting, components are involved: host immunity, HBV itself, and the factors that influence the host immunity or virus, such as NA and insulin resistance (IR) [1,3].

Nucleos(t)ide analogs, such as entecavir (ETV), potently suppress HBV DNA and, thus, prevent disease progression and reduce HCC development [1,2,5]. Aside from HBV DNA and alanine aminotransferase (ALT), hepatitis B surface antigen (HBsAg) or chemokines can help monitor the effects of NA on HBV and immunity [5,6,7]. Serum HBsAg was shown to predict disease progression and the risk of HCC in patients with low HBV loads [8,9]. In addition, quantitative HBsAg levels could predict HBsAg loss in hepatitis B e antigen (HBeAg)-positive patients whose HBsAg was diminished markedly during the first year of NA treatment and could predict an enduring off-NA response in HBeAg-negative patients with low HBsAg levels (<100 IU/mL) following consolidation therapy [5,7]. Additionally, interferon-γ-inducible protein of 10 kDa (IP-10), a pleiotropic chemokine derived from different immune and tissue cells, contributes to liver injury in HBV transgenic mice and is also an independent biomarker of post-liver transplantation hepatic fibrosis in hepatitis C virus (HCV) infection [10,11]. Similarly, the serum levels of IP-10 could also predict an HBsAg decline in HBeAg-negative CHB patients on ETV [6]. This network of cytokines and chemokines is essential not only for the cross-talk between different immune effectors but also for hepatic injury and regeneration [3,10,12].

Another vital factor, IR, triggers pro-inflammatory mediators such as interferon-γ, interleukin-6, and IP-10; it also polarizes macrophages and T cells preferentially to pro-inflammatory phenotypes [13,14]. Meanwhile, IR results in IR associated disorders (IRAD), including prediabetes, type 2 diabetes mellitus (T2DM), and fatty liver [15]. These metabolic diseases share a common pathophysiological pathway and a lot of clinical and epidemiological evidence supports the close links [16,17]. In North America, the prevalence of prediabetes and T2DM were 7.8% and 12.5% in the HBV population with a median age of 44 years; moreover, the prevalence reached 11% (prediabetes) and 24.4% (T2DM) in the subgroup of patients aged 50 years or over [18]. Allegedly, IR gives rise to hepatic and systemic inflammation, causing non-alcoholic fatty liver disease (NAFLD), autoimmune disease, and gut dysbiosis [13,19]. Moreover, the protective anti-HBsAg antibody level decreases considerably in non-diabetic adults with elevated IR [20]. In CHB with low levels of HBV DNA, an increased incidence of HCC and liver-related death is evident with IRAD [21]. Even after seroclearance of HBsAg, diabetes still leads to the occurrence of HCC [22]. Apparently, IR has detrimental effects on patients with CHB [21,22]. Other physical or pathological factors, such as aging or cachexia, also harness the immune system in complex ways [4,13]. In addition to the serum markers and image studies monitoring the hepatic sequelae, the non-invasive fibrosis-4 index (FIB-4) helps to detect liver fibrosis and estimate the risk of HCC in CHB [23,24].

Nonetheless, there have been few studies that directly address how IRAD interfere with long-term changes in HBsAg in a real-world cohort. On top of that, a debate about the relationships of HBV, IR, and NAFLD persists. Many studies have reported that HBV is not associated with IR or hepatic steatosis and even reduces the risk of NAFLD [25,26,27,28]. In contrast, much evidence argues that HBV carriers have significantly higher rates of IR, prediabetes, T2DM, and metabolic syndromes [18,29,30]. Notably, many chronic illnesses accompanied by CHB, such as chronic kidney disease (CKD), chronic inflammation-related anemia, and LC, could complicate the study of immunity and metabolism [4,31,32,33]. We aimed to clarify how IRAD affect the kinetics of HBsAg in patients with CHB under long-term ETV therapy and to evaluate the discrepancy between the groups with and without T2DM or prediabetes by investigating cytokine profiles and FIB-4 scores to elucidate the possible immune-metabolic mechanism.

## 2. Experimental Section

### 2.1. Study Design and Data Source

**Figure 1:**
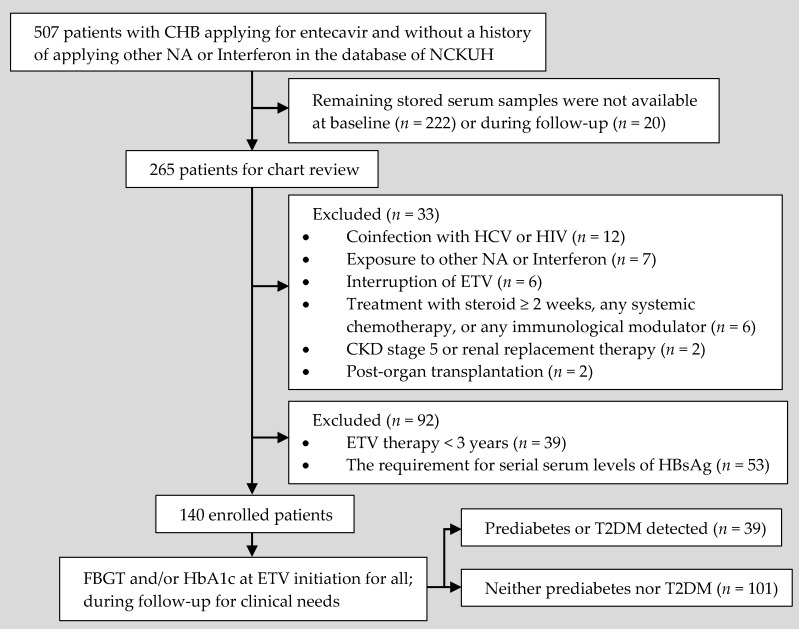
Patient disposition. CHB, chronic hepatitis B; CKD, chronic kidney disease; ETV, entecavir; FBGT, fasting blood glucose test; HbA1c, hemoglobin A1C; HBsAg, hepatitis B surface antigen; HCV, hepatitis C virus; HIV, human immunodeficiency virus; NA, nucleos(t)ide analogs; NCKUH, National Cheng Kung University Hospital; T2DM, type 2 diabetes mellitus.

The current study was a retrospective study in a tertiary medical center (Figure 1). We enrolled treatment-naive CHB patients who had received at least three years of consecutive ETV treatment at the National Cheng Kung University Hospital (NCKUH), Tainan, Taiwan. The enrolled patients began ETV therapy between December 2007 and January 2015, and the last follow-up ended between December 2012 and November 2017. The subjects were included only if they were over 20 years old, ETV was the only anti-HBV therapy administered without interruption, and the indications followed the treatment guideline [5]. All subjects required an Eastern Cooperative Oncology Group (ECOG) performance status of 0 or 1, and ECOG scores ≤1 over the study period. After the first treatment year (48 weeks after ETV initiation), subjects must have had at least two HBsAg quantifications in separate years, including two reports between the first and the sixth year, when the treatment time was less than 6 years or one data point before and another one after the sixth year when the treatment time was longer. The exclusion criteria were (1) prior treatment history with NA or interferon; (2) coinfection with HCV or human immunodeficiency virus infection; (3) CKD stage 5 or renal replacement therapy; (4) treatment with steroid ≥2 weeks, any systemic chemotherapy, or any immunological modulator and (5) post-organ transplantation. The study was conducted according to the Declaration of Helsinki. Informed consent was obtained at the request of the Institutional Review Board of NCKUH, which approved this study (A-ER-106-065, registered 23 February 2017).

### 2.2. Follow-Up and Data Collection

We reviewed the chart records for the patients’ anthropometrical characteristics. All enrolled subjects were followed up at three- to six-month intervals with liver biochemistry, HBeAg, HBV DNA, and abdominal sonography. Other baseline hematological and serum biochemistry data were determined at ETV initiation, including hemoglobin, platelet, creatinine, either fasting glucose or hemoglobin A1C, and either triglycerides or low-density lipoprotein cholesterol. These data must be checked once every other year but also within the first three years of active surveillance. If the initial and repeated data were within the normal ranges, the follow-up depended on the individual physicians. If there were any abnormal data points or new clinical manifestations, the relevant examinations were repeated for confirmation. For serial serum levels of HBsAg, we acquired the data of HBsAg at the baseline and yearly (within the established timeframe (any time between every 48 ± 12 weeks)); we allowed for missing data.

### 2.3. Terms and Definitions of Hepatic Events, Virology, Prediabetes, T2DM, and Other Chronic Comorbidities

Baseline data regarding age, sex, status of HBeAg, HBV genotype, HBV DNA, HBsAg, and ALT were all acquired at ETV initiation. Besides, we defined LC and HCC at the baseline, new HCC, and NA-treatment-related virological terminologies explicitly in Table S1. For either prediabetes or T2DM, reported data were within the whole study period (Tables S1 and S2). The other chronic comorbidities, namely, anemia, CKD, dyslipidemia, and advanced fatty liver (AFL), data were confirmed within the first 3 years of ETV treatment (Table S1). We acquired the components of FIB-4 at ETV initiation.

### 2.4. Laboratory Measurements

Serum HBsAg and HBV DNA level measurements followed the methods as previously described [5]. The upper limit of normal for ALT was 50 U/mL in males and 35 U/mL in females at NCKUH. Blood samples were collected and serum was prepared by nurses according to standard procedures. Blood was centrifuged (2000 revolutions per minute for 10 min at 4 °C), and serum was collected. All samples were stored at −80 °C until use. For the analysis of cytokines, independent laboratory staff studied all remaining stored serum samples collected after January 2014, at the baseline, first, third, and sixth year after the initiation of ETV therapy. The concentrations of IP-10 and the other nine types of cytokines regarding regulatory T cells and T helper cell type 1, 2, and 17 were measured using a cytometric bead array, Human Soluble Protein Flex Set System kit (BD Biosciences, San Diego, CA, USA), according to the manufacturer’s protocol. Data acquisition was performed using a FACSCanto II cytometer (BD Biosciences).

### 2.5. Statistical Analyses

Continuous variables are expressed as the mean ± standard deviation or the median (interquartile range), as appropriate. Categorical variables are presented as numbers (percentages). Statistical analysis was performed by Student t-test, one-way ANOVA, Mann–Whitney test, or Kruskal–Wallis test, for continuous parameters, and the chi-square test or Fisher’s exact test for categorical parameters, as appropriate. In the in-depth analysis, when the difference was statistically significant among the three subgroups, we then conducted post hoc analysis, including Dunn’s test after the Kruskal–Wallis test, Scheffe’s test after one-way ANOVA, and Bonferroni adjustment after Chi-Squared test or Fisher’s exact test. Our previous study indicated that the trajectory of HBsAg was similar one year after ETV therapy among the groups with different statuses of HBeAg and LC [5]. Hence, we distinguished the baseline HBsAg, which was determined ahead of ETV therapy, from the outcome variables, i.e., the subsequent annual HBsAg levels after ETV initiation.

Initially, we treated baseline variables and time points as categorical variables for depicting longitudinal changes of serum HBsAg levels in a linear mixed effects model (LMEM) with a random intercept. Afterwards, we considered time as a continuous variable and utilized a longitudinal LMEM to examine the effects of the 14 baseline variables, time, and interaction between baseline variables and time on HBsAg after one year of ETV treatment. For repeated measurements in a longitudinal study, LMEMs have been widely adopted in the management of individual heterogeneity [34,35,36]. The 14 baseline variables were age, sex, cirrhosis, HCC, HBeAg, HBV genotype, HBV DNA, HBsAg, ALT, anemia, CKD stage, prediabetes or T2DM, dyslipidemia, and AFL. No multicollinearity between the 14 factors were detected when significant multicollinearity was defined as a variance inflation factor >4. The LMEM used the SAS MIXED procedure with compound symmetry covariance structures to examine HBsAg changes over 9 years, from the second to the tenth year of ETV treatment. Initially, we tested interactions between baseline variables and time using bivariate analysis. Next, we included significant interaction terms and the 14 baseline variables into the final multivariate analysis. All data were analyzed with SAS software (version 9.4; SAS Institute Inc., Cary, NC, USA). All statistical tests were two-tailed, and a *p* value of <0.05 indicated statistical significance.

## 3. Results

### 3.1. Characteristics of the Enrolled Patients and Their Clinical Results

We enrolled 140 treatment-naive CHB patients who had received at least 3 years of ETV therapy from the previously published cohort [5]. In the current study, the mean age was 51.82 ± 11.55 years, and 93 (66.4%) were male. The median treatment time was 6.90 (4.47–9.01) years. Ninety-six (68.6%) patients were HBeAg-negative, and 46 (32.9%) were cirrhotic. Only 18 (12.9%) had HCC when they initiated ETV therapy. Thirty-nine (27.9%) patients were diagnosed as having prediabetes or T2DM, namely, 12 with prediabetes and 27 with T2DM. Most prediabetes or T2DM (23, 59.0%) occurred at the baseline, and the other 41% had a median of 1.85 (0.93–4.20) years. The persistence time was 5.21 (2.17–7.32) years for prediabetes and 7.17 (5.29–9.21) years for T2DM. The patients with prediabetes or T2DM were older, predominantly HBeAg-negative, with lower baseline HBsAg, and more dyslipidemia; nevertheless, the other comorbidities did not show significant differences (Table 1). Overall, 125 (89.3%) patients showed virological response. Of 44 HBeAg-positive patients, 23 (52.3%) achieved HBeAg clearance and 15 (34.1%) achieved HBeAg seroconversion. Only 8 (6.6%) patients were found to eventually acquire new HCC. No patient died nor experienced adverse events due to the drug by the end of this study (Table S2).

### 3.2. HBsAg Kinetics During Long-Term ETV: Prediabetes or T2DM Hindered the Fading of HBsAg over Time Independently and Markedly

**Figure 2:**
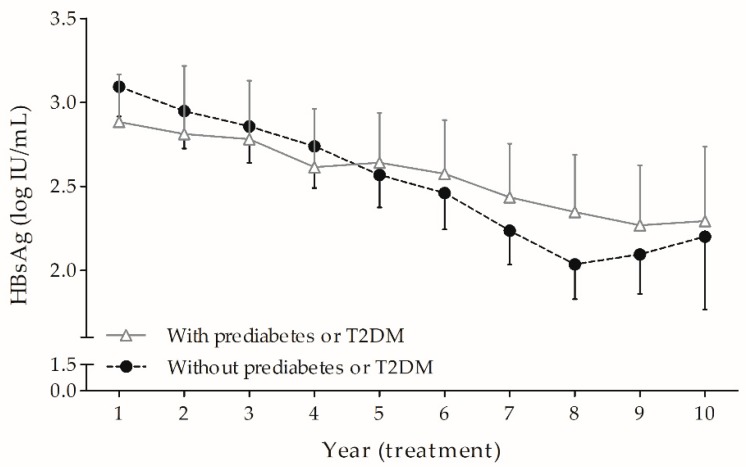
HBsAg kinetics during entecavir treatment over time, categorized by with or without prediabetes or T2DM. The serum HBsAg levels were derived from a linear mixed effects model, with adjusted mean and 95% confidence intervals (error bars). HBsAg, hepatitis B surface antigen; T2DM, type 2 diabetes mellitus.

In an LMEM where time points were considered categorical variables, we illustrated the decline of serum HBsAg levels over time in all 140 patients with or without prediabetes or T2DM (Figure 2). Next, in order to explore how baseline variables affected slopes of HBsAg trajectory, we regarded time as a continuous variable in a longitudinal LMEM in all 140 enrolled patients throughout the study periods. The interactions between baseline variables and time were tested using bivariate analysis, which indicated that age, prediabetes or T2DM, and AFL had significant interactions with time (Table S3). Then, the three significant interaction terms and the 14 baseline variables were included in the final multivariate analysis (Table 2). This analysis revealed that the patients with HBV genotype C (coefficient (standard error, SE) = 0.37 (0.13) log IU/mL, *p* = 0.007) and higher baseline HBsAg (coefficient (SE) = 0.55 (0.12), *p* < 0.0001) had higher serum HBsAg levels at the beginning of the second year of ETV treatment. Moreover, patients with prediabetes or T2DM showed a significant slower decline in serum HBsAg from the second to the tenth year (coefficient (SE) = 0.08 (0.02) log IU/mL/treatment time in year, *p* < 0.0001), and the same held true for those who were older (coefficient (SE) = 0.003 (0.001) log IU/mL/(treatment time in year × age in year), *p* = 0.001). In contrast, patients with AFL showed a faster decline in HBsAg during the period (coefficient (SE) = −0.07 (0.02) log IU/mL/treatment time in year, *p* = 0.001).

### 3.3. Cytokine Profiles: Higher IP-10 Levels in Patients with Prediabetes or T2DM at the Sixth Year of ETV Therapy

We acquired all remaining stored serum samples for the analysis of cytokine profiles in patients with or without prediabetes or T2DM. Only a cross-sectional comparison was available because of concerns regarding the decay of these molecules with time [37]. The serum IP-10 in the patients with prediabetes or T2DM at the sixth year was significantly higher than that in the ones without prediabetes or T2DM (*p* = 0.013) (Table 3; Figure S1). Specifically, the storage time of serum specimens between these two groups was similar, considering the cytokine degradation with time after the collection of serum. Except for IP-10, the level of other cytokines between these two groups at the indicated time points was not significantly different. Regardless of the lack of statistical significance, there were slightly lower levels of interleukin-10 and interferon-γ in the group with prediabetes or T2DM at the third year and the sixth year after ETV therapy, respectively.

### 3.4. FIB-4 Score: Highest in Subjects with T2DM in the In-Depth Analysis

We verified the baseline FIB-4 score and levels of other variables among the groups with prediabetes, T2DM, or neither of them. Six patients were excluded because they did not have platelet data at the baseline, and all of them were non-cirrhotic and without prediabetes or T2DM. When compared to those without prediabetes or T2DM, patients with T2DM were older and had higher FIB-4 scores, lower platelet counts, as well as a higher proportion of HBeAg negativity, HBV genotype B, dyslipidemia, and AFL at the baseline (Table 4).

## 4. Discussion

We demonstrated that the occurrence of prediabetes or T2DM is an independent factor hindering HBsAg decline in patients with CHB receiving long-term ETV. To the best of our knowledge, this is the first real-world cohort to be evaluated regarding how IRAD affect the kinetics of HBsAg directly. Additionally, FIB-4 scores at the baseline and levels of IP-10 at the sixth year of the therapy were both higher in the group with prediabetes or T2DM, which indicates advanced hepatic fibrosis and increased inflammation. However, in our patients with AFL, the reduction in HBsAg remains. This phenomenon indicates that IRAD act as a double-edged sword on the kinetics of HBsAg. IR impairs the immune system, which hinders the effective control of HBV and leads to a sluggish reduction in HBsAg [20,38,39]; conversely, but simultaneously, IR promotes hepatic fatty changes and inflammation and reduces the amount of viable liver parenchyma, along with the resident HBV, which results in overt HBsAg decreases [15,16,17].

Generally, HBsAg decline is less pronounced when the HBV DNA becomes undetectable after one or two years of NA therapy [5,7,40]. Because most of our patients achieved virological response within one year after ETV initiation, we distinguished the baseline HBsAg from the annual follow-up HBsAg values and did not factor the fluctuation of HBsAg over the first year in the long-term kinetics. Although the levels of HBsAg remained reliably different in the groups based on genotypes or baseline HBsAg, both variables neither interact with time nor affect the slopes significantly over time, which is consistent with our previous study regarding HBeAg status and LC one year after ETV initiation [5]. With regard to the virus–host interaction in the natural course of CHB, patients with genotype C tend to have more liver complications, such as cirrhosis and HCC, when compared to ones with genotype B [41,42]. As for antiviral treatments, HBV genotypes can be used to predict treatment response to interferon but seem to have no significant influence on NA therapy [41,42]. One study indicated that HBV genotypes B and C did not affect the antiviral response to lamivudine [43]; also, another study reported that HBV genotypes were not significantly associated with undetectable levels of HBV DNA one year after ETV therapy initiated [44]. In this study, HBV genotypes affected serum HBsAg levels at the beginning but did not affect long-term HBsAg decline during ETV therapy. To make a more convincing statement regarding the relationship between HBV genotypes and long-term NA therapy, further studies are warranted.

In contrast, IRAD, including prediabetes, T2DM, and AFL, strongly interact with time and alter the slopes, respectively; age also relatively affects the HBsAg slopes. The lower HBsAg serum levels indicate the higher immune control of HBV during the natural course of the disease; also, the reduction in HBsAg is a positively associated treatment response in those CHB patients receiving PegIFNa or NA [7]. Additionally, elderly patients have an unfavorable immunological profile, and the patients with prediabetes or T2DM tend to be older [4,22]. Therefore, it seems reasonable that prediabetes, T2DM, or older age slows the reduction in HBsAg over time. Nevertheless, hepatic steatosis could interfere with HBsAg expression, and the AFL predicts HBsAg seroclearance during CHB [45]. HBsAg decline seems to present in the opposite conditions, while the two conditions are not mutually exclusive: It is a good sign when the drop indicates good control of the virus by the host immunity [7]; in contrast, it is bad news if the decrease comes from poor liver reserves after hepatic fatty changes [19,20,38]. Previous studies support our explanation regarding the kinetics of HBsAg. Insulin resistance not only disturbs the immunological clearance of HBsAg but also contributes to hepatic steatosis and fibrosis that facilitates the loss of HBsAg. Both effects of IR pose known hazards to patients with CHB [3,21,22].

The supporting evidence is that IP-10, which is higher in the group with prediabetes or T2DM at the sixth year, attracts pro-inflammatory immune effectors to the liver, without antiviral activity against HBV [10,46]. In the meantime, IP-10 increases substantially in patients with NAFLD alone or with incident diabetes and could be used as a biomarker to predict fibrosis progression [46]. IP-10 can induce chemotaxis, apoptosis, and cell proliferation in response to infection or inflammatory diseases, and it relatively reflects the exuberant immune response [10,11]. One case-control study mentioned that lower levels of serum IP-10 were associated with HBsAg seroclearance; notably, IP-10 was the only significant factor among the investigated cytokines [47]. Allegedly, higher serum IP-10 (>350 pg/mL) at baseline predicts a larger HBsAg drop in patients on four years ETV [6]. Of note, although the studied population and stages of CHB were different, these data point to IP-10 as being indispensable and working in an exquisite way in the interplay of immunity and HBV [11,47]. Our study included important factors influencing immunity; furthermore, it is clear that prediabetes or T2DM tends to increase serum IP-10 levels and is associated with higher baseline FIB-4 scores, which is compatible with its role of long-standing harmful inflammation [4,13].

The disagreement about the relationship between HBV infection and IRAD could possibly be explained from an immune-metabolic virology perspective. Most studies rely on HBsAg seropositivity without clarifying the divergence of HBsAg kinetics or constraining essential factors affecting HBsAg, not only in the studies that reported no or negative correlation [26,27,28] but also in those stating a positive association [18,29,30]. However, our report suggests that IRAD affects HBsAg decline in the reverse direction, i.e., lessening directly or hastening indirectly, because of the diverse conditions; hence, a discrepancy may occur when the stages of CHB, degrees of hepatic steatosis and fibrosis, and other major immunological factors cannot be strictly controlled. Accordingly, a theoretical hypothesis of two conditions has been tentatively put forward for explanation (Figure S2).

Our study has several limitations. First, it focuses on a highly selected group of patients with CHB that received long-term ETV without overt event, and extrapolation should be prudent. The small size of patients with prediabetes or T2DM (*n* = 39) has also been noted. Nevertheless, the impact of IRAD on HBsAg is more evident when the suppressed HBV cannot interfere with immunity as significantly as HBV itself can in the natural course of the disease. Second, repeated blood sampling over the entire follow-up period was unavailable for all chronic diseases due to the retrospective design of the study. Nevertheless, the prevalence of prediabetes or T2DM in our cohort was similar to that in one large cohort study [18]. Furthermore, when compared with the general population, the prevalence of the defined dyslipidemia in this cohort (42.1%) was lower than that in Australia (63.2%) but similar to that in Philippines (47.2%, hyperlipidemia; 38.6%, hypertriglyceridemia) and Thailand (38.6%, hypertriglyceridemia) [48]. Notably, the different cut-off blood levels significantly affect the diagnosis and prevalence of dyslipidemia among studies [48]. Most large studies on CHB and chronic illness have used data at the baseline or within the initial periods; however, it deserves a long-term prospective design with successive blood draw. Third, the timing of detecting prediabetes or T2DM during follow-up was limited to clinical evaluation, and we did not consider other methods such as homeostasis model assessment of IR (HOMA-IR). For further studies in the future, HOMA-IR could be used for quantitative evaluation of IRAD. We should also note these blood tests only reflect the blood glucose ante cibum; continuous glucose monitoring is a better solution. Additionally, hepatogenous diabetes with false negative results is another concern [49].

Because the cytokine concentrations declined to noticeable levels after long-term storage [37], we only acquired serum stored less than five years for cytokine assessment. Therefore, the patients who received cytokine assessment were not identical among the treatment time-points (baseline and the first, third, or sixth year). To curtail bias, we compared the cytokine levels only cross-sectionally (prediabetes or T2DM vs. neither of them) rather than longitudinally. The serum storage time between the two groups were similar at each treatment time-point. Furthermore, the cytokine profiles were focused only on patients with CHB receiving long-term ETV therapy specifically to elucidate the discrepancy between the subgroups after the LMEM confirmed that IRAD did affect HBsAg decline. Although a network of cytokines and chemokines is crucial for HBV control, the cytokines or chemokines alone could not directly reflect the immunological activity [50,51]. Moreover, cytokine profiles could vary in different clinical scenarios among studies and we provided the Supplemental Information for reference (Table S4). It is necessary to heed these limitations and the research design to avert possible errors in ensuing studies.

## 5. Conclusions

In conclusion, we demonstrated that prediabetes or T2DM significantly hampered the decline in HBsAg, and this effect can be offset by advanced hepatic fatty changes. Furthermore, we elucidated the pro-inflammatory and fibrosis-prone mechanism regarding IP-10 at the sixth year and FIB-4 scores at baseline. How the immune system operates remains largely unanswered in CHB, but it is nevertheless possible to say what the composition of an answer must be. The surveillance of IRAD is worthwhile since it is detrimental to patients with CHB, and the immune system maintains this relentless inflammation. To reach a genuine truce, further studies of CHB should be devoted to the immune system and the key modulators, especially IRAD.

## Figures and Tables

**Table 1:** Clinical characteristics of the 140 enrolled patients, categorized by with prediabetes or type 2 diabetes mellitus (T2DM) or neither of them.

Characteristics	Total (*n* = 140)	Prediabetes or T2DM (*n* = 39)	Neither of Them (*n* = 101)	*p* Value
Age (year)	51.82 ± 11.55	57.74 ± 10.55	49.53 ± 11.14	**0.0001**
Male, n (%)	93 (66.4)	27 (69.2)	66 (65.3)	0.66
Treatment time (year)	6.90 (4.47–9.01)	7.03 (5.35–9.23)	6.83 (4.32–8.99)	0.57 ^c^
Cirrhosis, n (%)	46 (32.9)	15 (38.5)	31 (30.7)	0.38
HCC ^a^, n (%)	18 (12.9)	5 (12.8)	13 (12.9)	0.99
HBeAg negative, n (%)	96 (68.6)	34 (87.2)	62 (61.4)	**0.003**
HBV genotype ^b^: B vs. C, n (%)	64:57 (52.9:47.1)	21:11 (65.6:34.4)	43:46 (48.3:51.7)	0.09
HBV DNA (log IU/mL)	5.83 ± 1.74	5.57 ± 1.65	5.93 ± 1.77	0.27
HBsAg (log IU/mL)	3.18 ± 0.79	2.98 ± 0.58	3.26 ± 0.85	**0.032**
ALT (× ULN)	1.68 (1.00–3.57)	2.04 (1.04–3.77)	1.56 (0.98–3.37)	0.36 ^c^
Anemia, n (%)	22 (15.7)	5 (12.8)	17 (16.8)	0.56
CKD stage 2–4: yes vs. no, n (%)	35:105 (25:75)	12:27 (30.8:69.2)	23:78 (22.8:77.2)	0.33
Dyslipidemia, n (%)	69 (49.3)	28 (71.8)	41 (40.6)	**0.001**
Advanced fatty liver, n (%)	23 (16.4)	10 (25.6)	13 (12.9)	0.07

Bolded *p* values < 0.05. Continuous variables are expressed as the mean ± standard deviation or median (interquartile range), and categorical variables are expressed as numbers (percentages). ^a^ HCC diagnosed before or within half a year of entecavir therapy. ^b^ HBV genotype could not be determined in 19 patients (all HBeAg-negative, seven with and 12 without prediabetes or T2DM) because of low HBV viral loads in these patients. Only one patient without prediabetes or T2DM had a mixed genotype (B+C), and we took the mixed genotype as genotype C. ^c^ Mann–Whitney test, because the data did not fit a normal distribution. ALT, alanine aminotransferase; CKD, chronic kidney disease; HBeAg, hepatitis B e antigen; HBsAg, hepatitis B surface antigen; HBV, hepatitis B virus; HCC, hepatocellular carcinoma; ULN, upper limit of normal.

**Table 2:** Multivariate linear mixed effects models for predicting HBsAg during the 2nd to 10th years, including the 14 baseline variables and three significant interaction terms (age and time, prediabetes or type 2 diabetes mellitus (T2DM) and time, as well as advanced fatty liver and time).

Model Components	Multivariate Analysis (the General Model)		
	Estimate of Coefficient	Standard Error	*p* Value
Time (year)	−0.27	0.04	**<0.0001**
Baseline variables			
Age (year)	−0.00001	0.008	1.00
Sex (female vs. male ^a^)	−0.02	0.15	0.87
Cirrhosis (yes vs. no ^a^)	−0.19	0.17	0.29
HCC (yes vs. no ^a^)	−0.06	0.22	0.79
HBeAg (positive vs. negative ^a^)	0.27	0.18	0.14
HBV genotype (C vs. B ^a^)	0.37	0.13	**0.007**
HBV DNA (log IU/mL)	−0.04	0.07	0.58
HBsAg (log IU/mL)	0.55	0.12	**<0.0001**
ALT (× ULN): ≥2 vs. <2 ^a^	−0.28	0.16	0.07
Anemia (yes vs. no ^a^)	−0.31	0.19	0.10
CKD stage 2-4 (yes vs. no ^a^)	−0.15	0.15	0.30
Prediabetes or T2DM (yes vs. no ^a^)	0.01	0.18	0.94
Dyslipidemia (yes vs. no ^a^)	−0.14	0.14	0.32
Advanced fatty liver (yes vs. no ^a^)	0.18	0.23	0.44
Interaction terms			
Time × Age (year)	0.003	0.001	**0.0001**
Time × Prediabetes or T2DM (yes vs. no ^a^)	0.08	0.02	**<0.0001**
Time × Advanced fatty liver (yes vs. no ^a^)	−0.07	0.02	**0.001**
Intercept	1.66	0.61	**0.007**

Bolded *p* values < 0.05. ^a^ The latter value was taken as a reference. ALT, alanine aminotransferase; CKD, chronic kidney disease; HBeAg, hepatitis B e antigen; HBsAg, hepatitis B surface antigen; HBV, hepatitis B virus; HCC, hepatocellular carcinoma; ULN, upper limit of normal.

**Table 3:** Levels of the 10 kinds of cytokines/chemokines in the patients with “prediabetes or type 2 diabetes mellitus (T2DM)” or “neither of them” at the given time points.

	Baseline			1st Year			3rd Year			6th Year		
	Neither of Them (*n* = 18)	Prediabetes or T2DM (*n* = 4)	*p* Value	Neither of Them (*n* = 26)	Prediabetes or T2DM (*n* = 10)	*p* Value	Neither of Them (*n* = 40)	Prediabetes or T2DM (*n* = 17)	*p* Value	Neither of Them (*n* = 42)	Prediabetes or T2DM (*n* = 21)	*p* Value
IP-10	189.85 (134.15–249.17)	148.38 (123.95–177.69)	0.17	152.97 (124.88–206.46)	162.11 (119.00–210.40)	0.83	136.54 (111.99–179.11)	143.72 (130.23–199.41)	0.30	134.61 (94.30–176.84)	174.26 (156.12–200.77)	**0.013**
IFN-γ	16.32 (8.41–23.57)	13.41 (11.87–16.08)	0.67	11.05 (4.64–21.45)	13.11 (5.74–21.64)	0.92	13.31 (5.58–18.81)	8.02 (2.61–13.25)	0.11	7.86 (3.01–13.55)	4.82 (3.42–10.27)	0.36
TGF-β1	5295.80 (4245.30–5832.37)	5713.57 (4874.61–7310.43)	0.44	5836.38 (4534.38–7119.89)	5461.05 (4605.60–7601.74)	0.89	6172.21 (4524.55–7104.49)	5648.16 (4180.75–6996.47)	0.57	5704.12 (4553.30–7030.16)	5297.83 (4079.52–6514.09)	0.19
IL-1α	0.00 (0.00–0.25)	0.00 (0.00–0.40)	0.57	0.00 (0.00–0.44)	0.00 (0.00–0.95)	0.56	0.00 (0.00–0.00)	0.00 (0.00–0.00)	0.61	0.00 (0.00–0.00)	0.00 (0.00–0.05)	0.26
IL-4	0.54 (0.34–0.66)	0.33 (0.13–0.81)	0.35	0.24 (0.00–0.43)	0.23 (0.00–0.63)	0.80	0.00 (0.00–0.45)	0.00 (0.00–0.31)	0.70	0.00 (0.00–0.36)	0.00 (0.00–0.18)	0.30
IL-6	1.98 (0.74–3.79)	1.01 (0.16–1.43)	0.15	1.56 (0.77–3.83)	1.76 (1.35–3.20)	0.72	1.44 (0.88–2.42)	2.13 (1.21–3.90)	0.19	1.55 (0.70–2.61)	1.73 (1.29–3.49)	0.13
IL-10	0.78 (0.00–1.81)	0.45 (0.00–1.48)	0.54	0.00 (0.00–0.65)	0.58 (0.09–1.14)	0.08	0.57 (0.00–1.33)	0.63 (0.00–1.59)	0.87	0.77 (0.00–1.24)	1.03 (0.24–1.62)	0.12
IL-12p70	0.00 (0.00–0.23)	0.00 (0.00–1.01)	0.96	0.00 (0.00–0.21)	0.00 (0.00–0.51)	0.39	0.00 (0.00–0.53)	0.00 (0.00–0.25)	0.38	0.00 (0.00–0.61)	0.00 (0.00–0.21)	0.53
IL-17A	0.83 (0.00–1.66)	1.44 (0.39–3.09)	0.30	0.38 (0.00–0.76)	0.37 (0.19–1.13)	0.42	0.00 (0.00–0.71)	0.00 (0.00–0.95)	0.75	0.00 (0.00–0.67)	0.00 (0.00–0.58)	0.72
IL-21	0.00 (0.00–0.00)	0.00 (0.00–6.03)	0.75	0.00 (0.00–0.00)	0.00 (0.00–2.39)	0.56	0.00 (0.00–0.00)	0.00 (0.00–0.00)	0.33	0.00 (0.00–0.00)	0.00 (0.00–19.13)	0.23

Bolded *p* values < 0.05. Continuous variables are expressed as median (interquartile range). Mann–Whitney test. IFN, interferon; IL, interleukin; IP-10, interferon-γ-inducible protein of 10 kDa; IR, insulin resistance; TGF, transforming growth factor.

**Table 4:** In-depth analysis of the baseline fibrosis-4 index and relevant variables among the 134 patients, categorized by prediabetes, type 2 diabetes mellitus (T2DM), or neither of them.

Variable	Neither of Them (*n* = 95)	Prediabetes (*n* = 12)	T2DM (*n* = 27)	*p* Value	*Post hoc* Analysis
FIB-4	0.25 (0.16–0.40)	0.28 (0.20–0.62)	0.45 (0.26–0.76)	**0.007 ^b^**	T2DM > Neither of them ^b^
Age (year)	49.72 ± 11.33	54.61 ± 10.19	59.14 ± 10.60	**0.001 ^°^**	T2DM > Neither of them ^c^
Male, n (%)	62 (65.3)	9 (75.0)	18 (66.7)	0.87 ^d^	−
Treatment time (year)	6.74 (4.24–8.88)	6.18 (3.65–8.37)	7.47 (5.80–9.35)	0.18 ^b^	−
AST (× ULN)	1.16 (0.80–1.80)	1.00 (0.82–2.64)	1.57 (0.96–2.57)	0.35 ^b^	−
ALT (× ULN)	1.56 (0.96–3.34)	1.33 (1.01–3.52)	2.34 (1.04–3.92)	0.38 ^b^	−
Platelet (10^9^/L)	195.17 ± 66.89	165.00 ± 35.06	156.67 ± 58.67	**0.012 ^c^**	Neither of them > T2DM ^c^
Cirrhosis, n (%)	31 (32.6)	3 (25.0)	12 (44.4)	0.42 ^d^	−
HCC, n (%)	13 (13.7)	2 (16.7)	3 (11.1)	0.84 ^d^	−
HBeAg negative, n (%)	58 (61.1)	9 (75.0)	25 (92.6)	**0.007 ^d^**	T2DM > Neither of them ^d^
HBV genotype ^a^: B vs. C, n (%)	38:46 (45.2:54.8)	3:7 (30.0:70.0)	18:4 (81.8:18.2)	**0.003 ^d^**	T2DM > Neither of them; T2DM > Prediabetes ^d^ (% of genotype B)
HBV DNA (log IU/mL)	5.93 ± 1.76	6.24 ± 1.50	5.27 ± 1.65	0.15 ^c^	−
HBsAg (log IU/mL)	3.28 ± 0.82	3.18 ± 0.47	2.90 ± 0.61	0.07 ^c^	−
Anemia, n (%)	17 (17.9)	1 (8.3)	4 (14.8)	0.86 ^d^	−
CKD stage 2-4: yes vs. no, n (%)	20 (21.1)	2 (16.7)	10 (37.0)	0.21 ^d^	−
Dyslipidemia, n (%)	40 (42.1)	7 (58.3)	21 (77.8)	**0.004 ^d^**	T2DM > Neither of them ^d^
Advanced fatty liver, n (%)	12 (12.6)	0 (0.0)	10 (37.0)	**0.004 ^d^**	T2DM > Neither of them ^d^; T2DM > Prediabetes ^d^

Bolded *p* values < 0.05. Continuous variables are expressed in mean ± standard deviation or median (interquartile range), as appropriate. Categorical variables are expressed as numbers (percentages). ^a^ HBV genotype could not be determined in 18 patients (all HBeAg-negative, 11 without prediabetes or T2DM, two with prediabetes, and five with T2DM) because of low HBV viral loads in these patients. Only one patient without prediabetes or T2DM had mixed genotype (B+C), and we took the mixed genotype as genotype C. ^b^ Kruskal–Wallis test; Dunn’s test. ^c^ One-way ANOVA; Scheffe’s test. ^d^ Chi-Squared test or Fisher’s exact test, as appropriate; Bonferroni adjustment. ALT, alanine aminotransferase; AST, aspartate aminotransferase; FIB-4, fibrosis-4 index; HBeAg, hepatitis B e antigen; HBsAg, hepatitis B surface antigen; HBV, hepatitis B virus; HCC, hepatocellular carcinoma; ULN, upper limit of normal.

## Supplementary Materials

The following are available online at https://www.mdpi.com/2077-0383/8/11/1892/s1, Table S1: Terms and definitions, Table S2: Clinical events occurred during long-term entecavir therapy, Table S3: Bivariate linear mixed effect models for predicting HBsAg during the 2nd to 10th years, with regard to each variable and their interaction with time. Table S4: Levels of the different cytokines/chemokines in this study and other clinical scenarios, including healthy controls, diabetic patients, and patients with sepsis. Figure S1: Comparison of IP-10 levels between the groups categorized by with or without prediabetes or T2DM. Figure S2: A theoretical hypothesis of two conditions.
