# Supplementary material for: Insulin Resistance Associated Disorders Pivoting Long-Term Hepatitis B Surface Antigen Decline During Entecavir Therapy
Source: J Clin Med. 2019 Nov 6;8(11):1892. doi: 10.3390/jcm8111892 (PMC6912775; doi:10.3390/jcm8111892)
Supplement: Supplementary file 1 [file jcm-08-01892-s001.pdf]

Journal of
Clinical Medicine

MDPI

Table S1. Terms and definitions.

Term	Definition	Ref. S
LC	1. Time: LC was diagnosed before or within the first 3 years of ETV treatment.<br>2. Diagnosis: liver biopsy, imaging examinations [abdominal sonography, CT, or MRI], or clinical findings of portal hypertension (esophageal or cardiac varices by oesophagogastroduodenoscopy).	[1,2]
HCC	1. Time: HCC was diagnosed before or within the first half of a year of ETV treatment.<br>2. Diagnosis: histological examination (liver biopsy or surgery) or dynamic image studies (CT or MRI).	[2,3]
New HCC	1. Time: new HCC was diagnosed after half a year of ETV treatment in patients without a history of HCC.<br>2. Diagnosis: histological examination (liver biopsy or surgery) or dynamic image studies (CT or MRI).	[2]
Virological response	1. Time: the data have been evaluated during the entire follow-up period.<br>2. Diagnosis: the point at which serum HBV DNA level became undetectable (< 60 IU/mL) during treatment.	[2,4,5]
Virological breakthrough	1. Time: the data have been evaluated during the entire follow-up period.<br>2. Diagnosis: an increase in HBV DNA level of > 1 log10 IU/mL compared to the lowest value.	[4,5]
HBeAg seroclearance	1. Time: the data have been evaluated during the entire follow-up period.<br>2. Diagnosis: a loss of detectable HBeAg	[2,4,5]
HBeAg seroconversion	1. Time: the data have been evaluated during the entire follow-up period.<br>2. Diagnosis: a loss of detectable HBeAg and occurrence of anti-HBe	[2,4,5]
T2DM	1. Time: the data or information was based on, but not limited to, the active surveillance period (i.e. within the first 3 years of ETV treatment). Because the actual time of T2DM onset was often imperceptible, we included the data or information during the entire follow-up period and reported the time of diagnosis in Supplemental Table 2.<br>2. Diagnosis: (1) a known history of diabetes or current use of antidiabetic medications, or (2) fasting glucose ≥ 126 mg/dL, or (3) hemoglobin A1C ≥ 6.5%, or (4) a random plasma glucose ≥ 200 mg/dL and classic symptoms of hyperglycaemia or hyperglycaemic crisis.<br>3. Oral glucose tolerance test was not performed in this retrospective study.	[6]
Prediabetes	1. Time: the data or information was based on, but not limited to, the active surveillance period. Because the actual time of prediabetes onset was often imperceptible, we included the data or information during the entire follow-up	[6]

	period and reported the time of diagnosis in Supplemental Table 2.<br>2. Diagnosis: fasting glucose levels of 100-125 mg/dL or hemoglobin A1C of 5.7–6.4%.<br>3. If the patient had two separate events of impaired fasting glucose or the range of hemoglobin A1C (5.7–6.4%), we confirmed the diagnosis.	
Dyslipidemia	1. Time: the diagnosis was only based on the data or information within the active surveillance period.<br>2. Diagnosis: "diabetic dyslipidemia" was defined as (1) current use of lipid-lowering drug therapy, or (2) hyperlipidemia (low-density lipoprotein cholesterol ≥ 130 mg/dL), or (3) hypertriglyceridemia (triglycerides ≥ 150 mg/dL). In a subject without use of any lipid-lowering drug, we confirmed the diagnosis when the patient had two separate events of either hyperlipidemia or hypertriglyceridemia.<br>3. High-density lipoprotein cholesterol was not available in this retrospective cohort.	[7]
CKD	1. Time: the diagnosis was only based on the data or information within the active surveillance period.<br>2. Diagnosis: CKD was defined as abnormalities of kidney structure or function that was present for >3 months and was classified based on GFR category (stage 1 to 5). We excluded the patients with CKD stage 5 in this study. Therefore, the patients had either "no CKD or CKD stage 1" or "CKD stage 2, 3, or 4".<br>4. We reviewed the charts to exclude possible acute changes of the renal function.	[8]
Anemia	1. Time: the diagnosis was only based on the data or information within the active surveillance period.<br>2. Diagnosis: anemia was defined as hemoglobin < 12g/L in non-pregnant women and < 13 g/L in men.<br>3. We reviewed the charts to exclude possible acute changes, such as bleeding or major trauma.	[9]
Advanced fatty liver	1. Time: the diagnosis was only based on the data or information within the active surveillance period.<br>2. Diagnosis: advanced fatty liver was according to fatty liver echogenicity, and the severity of fatty liver echogenicity was graded as grade 0 to 3 (none, mild, moderate, and severe). We considered the moderate or severe fatty liver echogenicity as advanced fatty liver.<br>3. The abdominal ultrasonography was conducted by an independent radiologist or hepatologist every 3 to 6 months. We retrospectively evaluated the formal reports and the images for confirmation.	[10]
FIB-4	1. Time: the diagnosis based only on the data within the first year of the enrolment; we excluded the patients for FIB-4 analysis, if the data of platelet was missing.<br>2. The FIB-4 scores were calculated according to the equation: FIB-4 = age (years) × AST (U/L) ⁄ {platelet counts (10^9/L) × [ALT (U/L)]$^{1/2}$}.	[11,12]

NOTE. Active surveillance period: within the first 3 years of ETV treatment.

Abbreviations: ALT, alanine aminotransferase; Anti-HBe, anti-hepatitis B e antigen antibody; AST, aspartate aminotransferase; CKD, chronic kidney disease; CT, computed tomography; ETV, entecavir; FIB-4, fibrosis-4 index; GFR, glomerular filtration rate; HBeAg, hepatitis B e antigen; HBV, hepatitis B virus; HCC, hepatocellular carcinoma; IFG, impaired fasting glucose; LC, liver cirrhosis; MRI, magnetic resonance imaging; T2DM, type 2 diabetes mellitus.

Table S2. Clinical events occurred during long-term entecavir therapy.

Term	Patient number	Percentage (n/N)	Time (years)
Prediabetes (during the study period)	7	17.9% (7/39[b])	1.61 (0.84–3.40)
T2DM (during the study period)	9	23.1% (9/39[b])	2.09 (1.00–4.86)
Virological breakthrough[a]	9	6.4% (9/140)	n/a
Virological response	125	89.3% (125/140)	0.51 (0.25–0.96)
HBeAg clearance	23	52.3% (23/44[c])	2.62 (0.70–3.79)
HBeAg seroconversion	15	34.1% (15/44[c])	3.21 (0.51–3.88)
New HCC	8	6.6% (8/122[d])	6.75 (5.84–8.07)

NOTE. Time (years) is expressed as the median (interquartile range). No new liver cirrhosis was diagnosed during the entire study.

[a]Nine patients (6.4%) suffered from transient virological breakthrough once or twice separately, which subsided spontaneously only with close surveillance.

[b]Thirty-nine patients were diagnosed as having prediabetes or T2DM. When entecavir therapy initiated, 5 (12.8%) patients with prediabetes and 18 (46.2%) patients with T2DM were documented.

[c]Forty-four patients were HBeAg positive at baseline.

[d]Regarding the occurrence of new HCC, we excluded 18 patients who had HCC at baseline in the denominator.

Abbreviations: HBeAg, hepatitis B e antigen; HCC, hepatocellular carcinoma; T2DM, diabetes mellitus.

Table S3. Bivariate linear mixed effect models for predicting HBsAg during the 2nd to 10th years, with regard to each variable and their interaction with time.

Baseline variables	Bivariate analysis (each variable and the terms)		
	Estimate of coefficient	Standard error	P value
Age (year)	−0.03	0.01	**0.0002**
Time (year)	−0.29	0.04	<0.0001
Time × Age (year)	0.004	0.001	**<0.0001**

Intercept	4.44	0.35	<0.0001
Sex (female vs. male [a])	0.09	0.17	0.61
Time (year)	−0.12	0.01	<0.0001
Time × Sex (female vs. male [a])	0.02	0.02	0.15
Intercept	3.12	0.10	<0.0001
Cirrhosis (yes vs. no [a])	−0.32	0.16	0.06
Time (year)	−0.12	0.01	<0.0001
Time × Cirrhosis (yes vs. no [a])	0.02	0.02	0.12
Intercept	3.26	0.10	<0.0001
HCC (yes vs. no [a])	−0.28	0.23	0.24
Time (year)	−0.12	0.01	<0.0001
Time × HCC (yes vs. no [a])	0.001	0.021	0.97
Intercept	3.19	0.08	<0.0001
HBeAg (positive vs. negative [a])	0.66	0.16	**<0.0001**
Time (year)	−0.11	0.01	<0.0001
Time × HBeAg (positive vs. negative [a])	−0.02	0.02	0.19
Intercept	2.94	0.09	<0.0001
HBV genotype (C vs. B [a])	0.49	0.16	**0.003**
Time (year)	−0.12	0.01	<0.0001
Time × HBV genotype (C vs. B [a])	0.01	0.02	0.66
Intercept	2.97	0.11	<0.0001
HBV DNA (log IU/mL)	0.17	0.04	**0.0001**
Time (year)	−0.12	0.02	<0.0001
Time × HBV DNA (log IU/mL)	0.002	0.004	0.69
Intercept	2.17	0.26	<0.0001
HBsAg (log IU/mL)	0.59	0.08	**<0.0001**
Time (year)	−0.11	0.03	0.0002
Time × HBsAg (log IU/mL)	−0.0004	0.0092	0.96
Intercept	1.26	0.28	<0.0001
ALT (× ULN): ≥2 vs. <2 [a]	−0.10	0.16	0.55
Time (year)	−0.12	0.01	<0.0001

Time × ALT (× ULN): ≥2 vs. <2 [a]	0.02	0.02	0.27
Intercept	3.19	0.10	<0.0001
Anemia (yes vs. no [a])	−0.37	0.21	0.08
Time (year)	−0.12	0.01	<0.0001
Time × Anemia (yes vs. no [a])	0.02	0.02	0.31
Intercept	3.21	0.08	<0.0001
CKD stage 2-4 (yes vs. no [a])	−0.09	0.18	0.61
Time (year)	−0.11	0.01	<0.0001
Time × CKD stage 2-4 (yes vs. no [a])	−0.03	0.02	0.11
Intercept	3.17	0.09	<0.0001
Prediabetes or T2DM (yes vs. no [a])	−0.27	0.17	0.13
Time (year)	−0.14	0.01	<0.0001
Time × Prediabetes or T2DM (yes vs. no [a])	0.06	0.02	**<0.0001**
Intercept	3.23	0.09	<0.0001
Dyslipidemia (yes vs. no [a])	0.12	0.16	0.46
Time (year)	−0.11	0.01	<0.0001
Time × Dyslipidemia (yes vs. no [a])	−0.01	0.02	0.36
Intercept	3.10	0.11	<0.0001
Advanced fatty liver (yes vs. no [a])	−0.27	0.20	0.18
Time (year)	−0.10	0.01	<0.0001
Time × Advanced fatty liver (yes vs. no [a])	−0.07	0.02	**0.0002**
Intercept	3.18	0.08	<0.0001

NOTE. Bolded P values represent < 0.05, except for items that are all significant, namely, time (year) and intercept.

[a] The latter value was taken as reference.

Abbreviations: ALT, alanine aminotransferase; CKD, chronic kidney disease; HBsAg, hepatitis B surface antigen; HCC, hepatocellular carcinoma; T2DM, diabetes mellitus; ULN, upper limit of normal

Journal of Clinical Medicine

MDPI

Table S4. Levels of the different cytokines/chemokines in this study and other clinical scenarios, including healthy controls, diabetic patients, and patients with sepsis.

	**CHB on ETV without prediabetes or type 2 DM (6th year)**	**CHB on ETV with prediabetes or type 2 DM (6th year)**	**Healthy control**	**Healthy control**	**Healthy control**	**Healthy control**	**Type 2 DM**	**SFTS**	**Sepsis (survivor)**	**Sepsis (non-survivor)**
Supplemental reference	-	-	[13]	[14]	[15]	[16]	[13]	[14]	[17]	[17]
Patient number	42	21	23	38	37	66	25	50	31	29
Numeric expression	Median (IQR)	Median (IQR)	Median (IQR)	Median (IQR)	Median (Range)[a]	Median (Range)[b]	Median (IQR)	Median (IQR)	Median (IQR)	Median (IQR)
IP-10	134.61 (94.30–176.84)	174.26 (156.12–200.77)	18.12 (15.2–27.1)	65.1 (37.6–168.5)	10.6 (0.0–49.2)	32.24 (5.90–637.00)	30.8 (23.0–149.6)	2718.7 (1389.9–5278.9)	NA	NA
IFN-γ	7.86 (3.01–13.55)	4.82 (3.42–10.27)	4.12 (0.001–12.6)	2.2 (1.7–2.6)	0.0 (0.0–20.3)	8.68 (0.60–124.00)	12.03 (7.1–19.3)	10.8 (4.6–34.5)	12.34 (0.00–91.69)	28.71 (0.00–122.94)
TGF-β1	5704.12 (4553.30–7030.16)	5297.83 (4079.52–6514.09)	NA	NA	NA	NA	NA	NA	NA	NA
IL-1α	0.00 (0.00–0.00)	0.00 (0.00–0.05)	3.49 (2.45–5.2)	NA	NA	0.00 (0.40–1.40)	11.33 (3.6–24.9)	NA	NA	NA
IL-1β	NA	NA	16.47 (11.4–30.3)	NA	12.1 (0.0–46.6)	0.00 (0.02–0.70)	48.98 (33.8–76.6)	NA	0.39 (0.00–3.04)	1.30 (0.22–7.21)
IL-4	0.00 (0.00–0.36)	0.00 (0.00–0.18)	7.09 (2.3–20.6)	NA	33.6 (22.0–53.0)	0.00 (0.01–3.00)	29.63 (15.3–78.5)	NA	0.00 (0.00–0.03)	0.84 (0.00–26.28)
IL-6	1.55 (0.70–2.61)	1.73 (1.29–3.49)	8.43 (0.44–21.18)	4.0 (3.3–6.6)	0.0 (0.0–19.0)	0.00 (0.02–9.00)	239.41 (31–1018)	28.1 (10.7–70.5)	1957.77 (971.92–6295.47)	6254.96 (2446.01–15972.40)
IL-10	0.77 (0.00–1.24)	1.03 (0.24–1.62)	2.74 (1.89–5.29)	1.0 (0.8–1.7)	0.0 (0.0–0.0)	0.00 (0.10–2.00)	9.6 (4.62–16.27)	1228.4 (88.8–3960.9)	9.70 (2.00–40.89)	26.92 (5.29–96.58)
IL-12	NA	NA	NA	NA	76.2 (44.0–118.8)	NA	NA	NA	1.09 (0.00–40.89)	1.04 (0.00–6.79)

IL-12p70	0.00 (0.00–0.61)	0.00 (0.00–0.21)	12.03 (6.7–15.4)	NA	NA	0.00 (0.10–6.00)	22.92 (16.4–30.8)	NA	NA	NA
IL-17	NA	NA	NA	NA	22.8 (0.0–71.3)	0.00 (0.22–31.00)	NA	NA	0.00 (0.00–0.00)	0.00 (0.00–0.00)
IL-17A	0.00 (0.00–0.67)	0.00 (0.00–0.58)	0.001 (0.001–0.97)	NA	NA	NA	4.11 (1.05–8.47)	NA	NA	NA
IL-21	0.00 (0.00–0.00)	0.00 (0.00–19.13)	NA	NA	NA	NA	NA	NA	NA	NA

NOTE.

[a]In the supplemental reference [15], the range referred to minial and maximal values.

[b]In the supplemental reference [16], the range referred to values that were observed in extrapolated data.

Abbreviations: CHB, chronic hepatitis B; DM, diabetes mellitus; ETV, entecavir; IFN, interferon; IL, interleukin; IP-10, interferon-γ-inducible protein of 10 kDa; IQR, interquartile range; NA, not available; SFTS, severe fever with thrombocytopenia syndrome; TGF, transforming growth factor.

Journal of
Clinical Medicine
MDPI

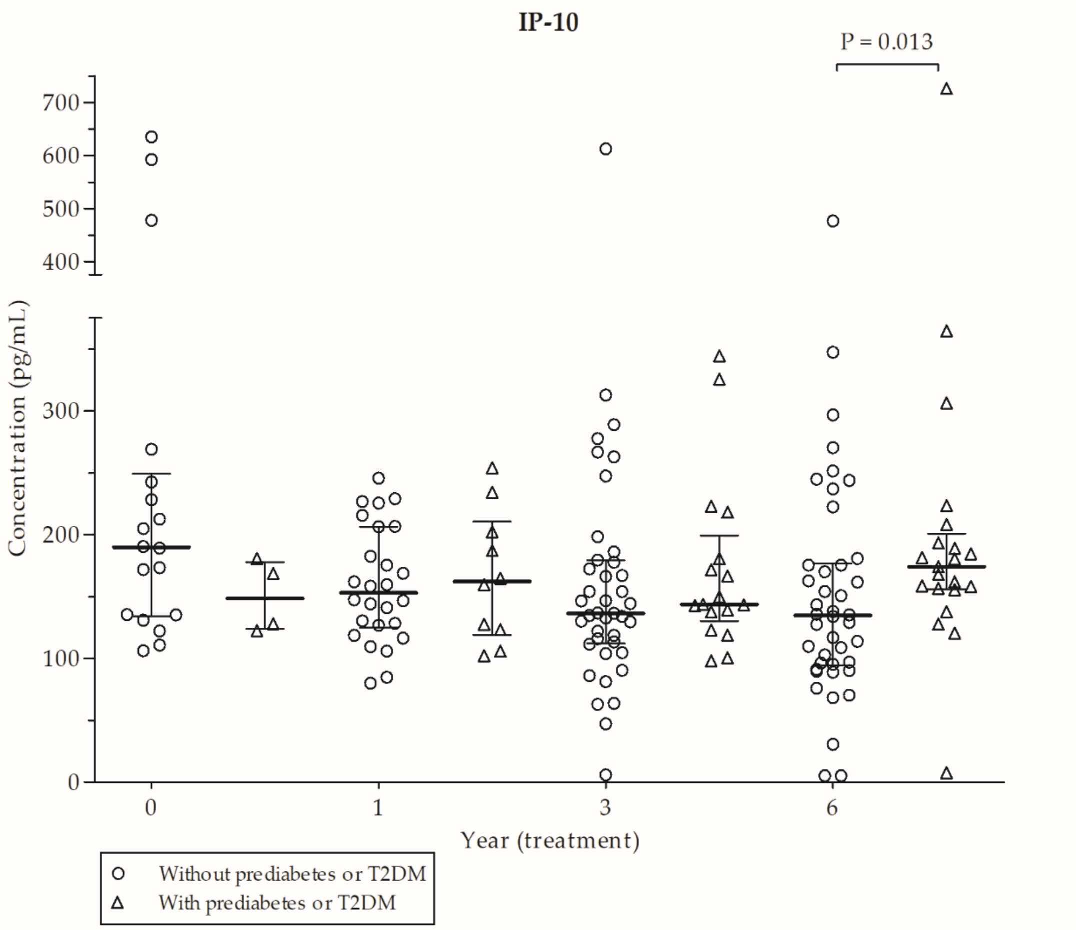

Figure S1. Comparison of IP-10 levels between the groups categorized by with or without prediabetes or T2DM. The lines indicate the median with the interquartile range. IP-10, interferon-γ-inducible protein of 10 kDa; T2DM, type 2 diabetes mellitus.

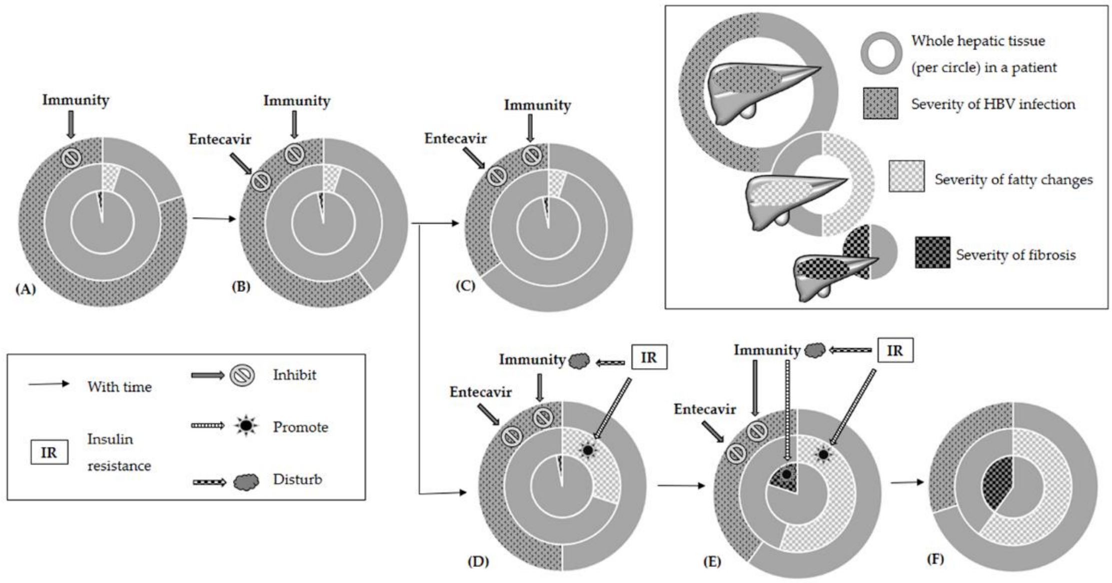

Figure S2. A theoretical hypothesis of two conditions. From (A) to (C), the combination of the immune response and entecavir suppressed but did not eradicate HBV; therefore, serum HBsAg levels decreased steadily but slowly. (D) When IR occurs, it promotes hepatic fatty changes and disturbs the

immune function, which is evident from elevated IP-10 levels; meanwhile, IR hindered the HBsAg decline (panel C and D compared). From (E) to (F), the IR is sustained and the advanced fatty liver occurs. Furthermore, an inflammation-prone immune system promoted fibrosis, as evidenced from the elevated fibrosis-4 index. Once the liver parenchyma, where HBV resides, shrinks significantly, the downswing of HBsAg accelerates again. The events in panels D, E, and F could occur simultaneously. HBsAg, hepatitis B surface antigen; HBV, hepatitis B virus; IP-10, interferon-γ-inducible protein of 10 kDa; IR, insulin resistance.

Supplemental references (Ref. S**)**

1. Tsochatzis EA, Bosch J, Burroughs AK. Liver cirrhosis. *The Lancet.* **2014**, *383*, 1749-61.
2. Lin TC, Chiu YC, Chiu HC, Liu WC, Cheng PN, Chen CY, Chang TT, Wu IC. Clinical utility of hepatitis B surface antigen kinetics in treatment-naive chronic hepatitis B patients during long-term entecavir therapy. *World J Gastroentero*. **2018**, *24*, 725-36.
3. European Association for the Study of the Liver. EASL Clinical Practice Guidelines: Management of hepatocellular carcinoma. *J Hepatol*. **2018**, *69*, 182-236.
4. Lampertico P, Agarwal K, Berg T, Buti M, Janssen HLA, Papatheodoridis G, Zoulim F, Tacke F. EASL 2017 Clinical Practice Guidelines on the management of hepatitis B virus infection. *J Hepatol*. **2017**, *67*, 370-98.
5. Sarin SK, Kumar M, Lau GK, Abbas Z, Chan HLY, Chen CJ, Chen DS, Chen HL, Chen PJ, Chien RN, et al. Asian-Pacific clinical practice guidelines on the management of hepatitis B: a 2015 update. *Hepatol Int*. **2016**, *10*, 1-98.
6. American Diabetes Association. 2. Classification and Diagnosis of Diabetes: Standards of Medical Care in Diabetes-2019. *Diabetes Care*. **2019**, *42*, S13-S28.
7. Jellinger PS, Handelsman Y, Rosenblit PD, Bloomgarden ZT, Fonseca VA, Garber AJ, Grunberger G, Guerin CK, Bell DSH, Mechanick JI, et al. American Association of Clinical Endocrinologists and American College of Endocrinology Guidelines for Management of Dyslipidemia and Prevention of Cardiovascular Disease. *Endocr Pract*. **2017**, *23*, 1-87.
8. Stevens PE, Levin A, Kidney Disease: Improving Global Outcomes Chronic Kidney Disease Guideline Development Work Group Members. Evaluation and management of chronic kidney disease: synopsis of the kidney disease: improving global outcomes 2012 clinical practice guideline. *Ann Intern Med*. **2013**, *158*, 825-30.
9. Stauder R, Valent P, Theurl I. Anemia at older age: etiologies, clinical implications, and management. *Blood*. **2018**, *131*, 505-14.
10. Saadeh S, Younossi ZM, Remer EM, Gramlich T, Ong JP, Hurley M, Mullen KD, Cooper JN, Sheridan MJ. The utility of radiological imaging in nonalcoholic fatty liver disease. *Gastroenterology*. **2002**, *123*, 745-50.
11. Tseng TC, Liu CJ, Su TH, Yang WT, Chen CL, Yang HC, Wang CC, Kuo SF, Liu CH, Chen PJ, et al. Fibrosis-4 Index Helps Identify HBV Carriers With the Lowest Risk of Hepatocellular Carcinoma. *Am J Gastroenterol*. **2017**, *112*, 1564-74.
12. Xiao G, Yang J, Yan L. Comparison of diagnostic accuracy of aspartate aminotransferase to platelet ratio index and fibrosis-4 index for detecting liver fibrosis in adult patients with chronic hepatitis B virus infection: a systemic review and meta-analysis. *Hepatology*. **2015**, *61*, 292-302.
13. Randeria, S. N.;Thomson, G. J. A.;Nell, T. A.;Roberts, T.; Pretorius, E. Inflammatory cytokines in type 2 diabetes mellitus as facilitators of hypercoagulation and abnormal clot formation. *Cardiovasc Diabetol*. **2019**, *18*, 72.
14. Liu, M. M.;Lei, X. Y.;Yu, H.;Zhang, J. Z.; Yu, X. J. Correlation of cytokine level with the severity of severe fever with thrombocytopenia syndrome. *Virol J*. **2017**, *14*, 6.
15. Arnaud, L.;Gorochov, G.;Charlotte, F.;Lvovschi, V.;Parizot, C.;Larsen, M.;Ghillani-Dalbin, P.;Hervier, B.;Kahn, J. E.;Deback, C., et al. Systemic perturbation of cytokine and chemokine networks in Erdheim-Chester disease: a single-center series of 37 patients. *Blood*. **2011**, *117*, 2783-90.
16. Chapman, P.;Reyes, C.; Gupta, V. Normal Physiological Levels of Human Cytokines Using Bio-Plex Pro™ Cytokine Assays. **2010**. Available online: https://www.bio-rad.com/webroot/web/pdf/lsr/literature/Bulletin_6029.pdf (accessed on 16, September, 2019).

17. Bozza, F. A.;Salluh, J. I.;Japiassu, A. M.;Soares, M.;Assis, E. F.;Gomes, R. N.;Bozza, M. T.;Castro-Faria-Neto, H. C.; Bozza, P. T. Cytokine profiles as markers of disease severity in sepsis: a multiplex analysis. *Crit Care*. **2007**, *11*, R49.

© 2019 by the authors. Submitted for possible open access publication under the terms and conditions of the Creative Commons Attribution (CC BY) license (http://creativecommons.org/licenses/by/4.0/).
